# Functional diversity of HIV-1 envelope proteins expressed by contemporaneous plasma viruses

**DOI:** 10.1186/1742-4690-5-23

**Published:** 2008-02-29

**Authors:** Tamara Nora, Francine Bouchonnet, Béatrice Labrosse, Charlotte Charpentier, Fabrizio Mammano, François Clavel, Allan J Hance

**Affiliations:** 1 Unité de Recherche Antivirale, INSERM U 552, Université Denis Diderot Paris 7, Paris F-75018, France

## Abstract

**Background:**

Numerous studies have shown that viral quasi-species with genetically diverse envelope proteins (Env) replicate simultaneously in patients infected with the human immunodeficiency virus type 1 (HIV-1). Less information is available concerning the extent that envelope sequence diversity translates into a diversity of phenotypic properties, including infectivity and resistance to entry inhibitors.

**Methods:**

To study these questions, we isolated genetically distinct contemporaneous clonal viral populations from the plasma of 5 HIV-1 infected individuals (n = 70), and evaluated the infectivity of recombinant viruses expressing Env proteins from the clonal viruses in several target cells. The sensitivity to entry inhibitors (enfuvirtide, TAK-799), soluble CD4 and monoclonal antibodies (2G12, 48d, 2F5) was also evaluated for a subset of the recombinant viruses (n = 20).

**Results:**

Even when comparisons were restricted to viruses with similar tropism, the infectivity for a given target cell of viruses carrying different Env proteins from the same patient varied over an approximately 10-fold range, and differences in their relative ability to infect different target cells were also observed. Variable region haplotypes associated with high and low infectivity could be identified for one patient. In addition, clones carrying unique mutations in V3 often displayed low infectivity. No correlation was observed between viral infectivity and sensitivity to inhibition by any of the six entry inhibitors evaluated, indicating that these properties can be dissociated. Significant inter-patient differences, independent of infectivity, were observed for the sensitivity of Env proteins to several entry inhibitors and their ability to infect different target cells.

**Conclusion:**

These findings demonstrate the marked functional heterogeneity of HIV-1 Env proteins expressed by contemporaneous circulating viruses, and underscore the advantage of clonal analyses in characterizing the spectrum of functional properties of the genetically diverse viral populations present in a given patient.

## Background

The population of human immunodeficiency virus type 1 (HIV-1) present in a single infected patient at any given time can show remarkable diversity. Moreover, the extent of diversity can evolve over time and is different in different genes. The most striking changes in diversity occur in the envelope glycoproteins (Env). The initial transmission of HIV-1 can result in infection of the new host with multiple viruses expressing genetically diverse *env *sequences [1-6]. Early in the evolution of infection, however, viruses expressing extremely homeogeneous *env *sequences become dominant, presumably reflecting the selection of viruses that are best adapted for replication in available target cells, and/or resistant to the nascent host immune response [1-3,7]. This initial homogenization is followed by a period often lasting many years, in which both the diversity of the *env *sequences and the evolutionary distance from the initially dominant strain increase linearly by approximately 1% per year [5,8-17]. Subsequently, the extent of viral diversity begins to plateau and, in the late stages of disease, a decline in viral diversity can be observed [8,11,12,18].

Although genetic diversity of the viral *env *has been extensively studied, less information is available concerning the extent that these genetically diverse Env proteins also display functional diversity. Envelope sequences have been amplified from plasma or short-term cell cultures and used to produce recombinant or pseudotyped viruses expressing primary *env *sequences [19-25]. Most studies have found that only 40–70% of such viruses are infectious, but quantitative assessment of the replicative capacity of a large number of viruses expressing different envelope sequences from a single patient has not been reported.

It also remains unclear the extent to which other properties of the viral Env proteins are shared by coexisting quasi-species from a given patient. Viral isolates obtained from different individuals can differ in their sensitivity to inhibition by chemokines [26-30], entry inhibitors [31-37], certain monoclonal antibodies [32,38], and autologous serum [26,39], but the extent that different viruses obtained from the same individual show similar sensitivity to a given entry inhibitor has not been extensively evaluated. Furthermore, replicative capacity, per se, can influence the sensitivity of viruses to inhibitors of entry [26,31,36,40], but it remains unknown whether or not the sensitivity of viruses from a given patient to entry inhibitors correlates closely with replicative capacity.

We have recently described an approach that allows the direct isolation of contemporaneous clonal viruses from the plasma of infected individuals, including viruses capable of using CCR5 and/or CXCR4 viral coreceptors [41,42]. These viruses are potentially useful for the evaluation of the functional correlates of *env *genetic diversity. First, each clonal virus emerges independently, and therefore viruses with low infectivity are not lost through competition with rapidly replicating viruses. Furthermore, the *env *sequences expressed by these viruses are genetically diverse, and the functional properties have not been modified by through mutation or recombination occurring during PCR. In this study, we have created recombinant viruses expressing Env proteins from these clonal viruses in a reporter construct expressing luciferase activity, and evaluated: i) the spectrum of infectivity observed for Env proteins expressed by contemporaneous viral clones from the same patient, ii) the ability of these viruses to infect different target cells, and, iii) the relationship between infectivity and the susceptibility of the Env proteins to several different entry inhibitors.

## Results

### Diversity of envelope sequences

**Figure 1:**
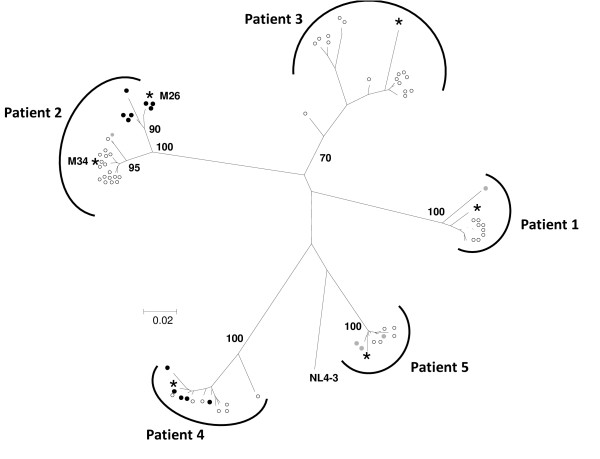
**Phylogenetic tree of envelope sequences of clonal viruses**. Shown is the neighbor-joining phylogenetic tree for nucleotide sequences coding the region of *env *encompassing C1 to V2 (corresponding to nucleotides 6440 – 6809 of HXB2) of the 70 clonal viruses evaluated in the study (open circles, strict R5 tropism, solid black circles, strict X4 tropism; solid gray circles, dual tropism), the consensus sequence of the same region for viral RNA amplified by RT-PCR from an aliquot of the plasma sample used to generate the clonal viruses (stars), and the sequence of the laboratory strain pNL4-3. Bootstrap values are also indicated. For patient 2, the consensus sequence of plasma viruses grouped with clonal viruses with X4 tropism at M26, and with clonal viruses with R5 tropism at M34.

Phylogenetic analysis indicated that *env *sequences (C1-V2 region) for all clones from each patient clustered together along with the consensus sequence obtained for bulk envelope sequences amplified directly from plasma by RT-PCR (Fig. 1). Viruses with both R5 and X4 tropism (see below) were isolated from 4 patients. For patients 1 and 2, sequences for viruses with R5 tropism appeared to be phylogenetically distinct from those of viruses with X4 (patient 2) or dual (patient 1) tropism, and the sequence diversity among viruses with similar tropism was lower than that of the entire viral population. In contrast, for patients 4 and 5, envelope sequences in the V1-V2 region for viruses with R5 tropism were not segregated from those of viruses with X4 (patient 4) or dual (patient 5) tropism. For patient 2, the consensus *env *sequence for plasma viruses clustered with the sequences of viruses with X4 tropism at month 26, but clustered with the sequences of viruses with R5 tropism at month 34.

The nucleotide diversity of *env *sequences extending from V1 to the middle of V4, calculated by the method of Tajima-Nei, ranged from 0.018 – 0.060, values typical of those obtained for sequences amplified from plasma by RT-PCR. The greatest diversity was observed for patient 3, despite that all viruses from this patient showed strict R5 tropism.

### Infectivity of recombinant viruses carrying primary envelope sequences in U373-R5 and U373-X4 target cells

**Figure 2:**
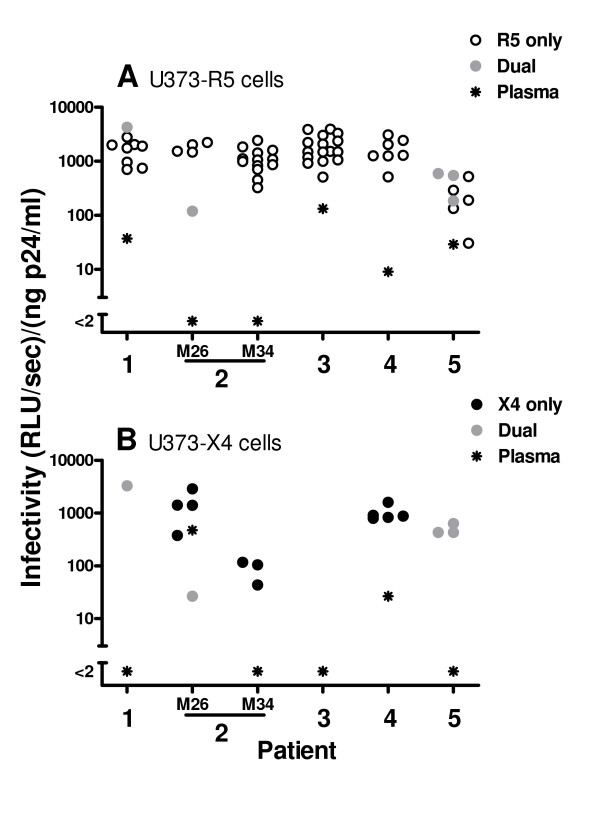
**Infectivity and co-receptor usage of envelope proteins expressed by clonal viruses**. Recombinant reporter viruses were generated in which the *env *sequence (gp120 + the extracellular domain of gp41) was derived from clonal viruses isolated from plasma of patients chronically infected with HIV-1. The ability of these viruses, which express *Renilla *luciferase in the place of Nef, to infect U373 cells stably expressing CD4 and either CCR5 (panel A) or CXCR4 (panel B) co-receptors was measured by evaluating luciferase expression in target cells 44 hours after infection. For patient 2, clonal viruses were obtained from plasma samples obtained 8 months apart (26 and 34 months after initial diagnosis). The ability of all recombinant viruses to infect the two cell types was evaluated, but results are shown only for viruses that induced significant luciferase activity in the indicated target cell type [>2 (RLU/sec)/(ng p24/ml)]. Viruses could be classified as having strict R5 tropism (open symbols) strict X4 tropism (solid black symbols) and dual tropism (sold gray symbols). Results for viruses expressing *env *sequences amplified by RT-PCR from patient plasma is also shown (stars). Each symbol is the mean of at least 3 independent experiments; the mean coefficient of variation for these results is as follows: U373-R5 cells, 42% (range 3 – 83%); U373-X4 cells, 50% (range 10 – 78%). For each patient, significant differences were found comparing the viruses with the highest and lowest infectivity (p < 0.05 – 0.001 by t-test).

To explore the functional capacities of Env proteins expressed by these clonal viruses, we generated recombinant reporter viruses in which the *env *sequence (gp120 + the extracellular domain of gp41) was derived from the different clonal viruses, and evaluated the ability of these luciferase-expressing viruses to infect U373 cells stably expressing CD4 and either CCR5 or CXCR4 co-receptors. All of the 70 recombinant viruses were infectious (Fig. 2). Viruses with strict R5 tropism were identified in all patients (n = 53), but clones with R5X4 tropism (n = 5) and/or strict X4 tropism (n = 12) were also identified in 4 of the 5 patients studied. The infectivity of dual-tropic viruses tended to be similar in the U373-R5 and U373-X4 target cells (compare gray symbols in Figs. 2A and 2B). Without exception, the infectivity of clones with R5 tropism was at least 5-fold lower than the infectivity of recombinant viruses carrying the Env from the laboratory-adapted strain NL-AD8 [mean infectivity in U373-R5 cells: 22524 (RLU/sec)/(ng p24/ml)]. The infectivity of some clones with X4 tropism was equivalent to that observed for recombinant viruses carrying the Env from pNL4-3 [mean infectivity in U373-X4 cells: 2650 (RLU/sec)/(ng p24/ml)].

Considerable variability was observed in the infectivity of viruses carrying different *env *sequences from the same patient. When U373-R5 cells were used as targets, the difference in infectivity between the most and least infectious viruses from each of the 5 patients averaged 1.0 ± 0.26 log_10 _(range 0.8 – 1.3 log_10 _difference). Some inter-patient variability in infectivity was also observed. When considered as a group, no significant differences in the infectivity of clones carrying Env proteins from patients 1–4 were observed, but the infectivity of clones from each of these patients was significantly greater than that of clones from patient 5 (p < 0.05 for all comparisons).

### Impact of the intracellular portion of gp41 on viral infectivity

**Figure 3:**
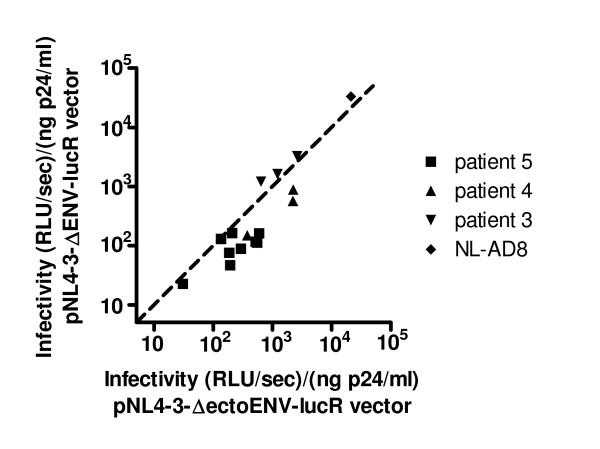
**The effect of the origin of the intracellular portion of gp41 on infectivity**. For selected clonal viruses isolated from patient 3 (inverted triangles), patient 4 (triangles), patient 5 (squares) or from the laboratory-adapted strain NL-AD8 (diamond), two types of recombinant reporter viruses were generated, one in which gp120 + only the extracellular domain of gp41 was derived from the clonal virus (pNL4-3-ΔectoENV-lucR, abscissa), and one in which gp120 and all of gp41 was derived from the clonal virus (pNL4-3-ΔENV-lucR vector, ordinate). The ability of these viruses to infect U373-R5 cells was compared by evaluating luciferase expression in the target cells 44 hours after infection. The infectivity of each pair of viruses was evaluated in three independent experiments, and each symbol represents the mean of these determinations. The dotted line is the line of identity. The correlation coefficient (Spearman) for the data shown is 0.86 (p < 0.0001).

The intracellular portion of gp41 is known to interact with Gag. To avoid potential incompatibilities between these viral proteins [43,44], we initially evaluated the infectivity of recombinant viruses in which both the intracellular portion of gp41 and Gag were derived from the pNL4-3 viral strain. Changes in the intracellular domain of gp41, however, have also been reported to influence Env function [45-48], and it was important to evaluate the possibility that incompatibilities between the extracellular domains of Env in some of the viruses and the intracellular domain of gp41 from pNL4-3 contributed to the wide range of infectivities observed. To do so, we compared the infectivity of selected recombinant viruses in which the intracellular domain of gp41 was derived either from pNL4-3 or from the primary viral isolate. As shown in Fig. 3, the infectivities of the two constructs were strongly correlated (Spearman r = 0.86; p < 0.0001). Viruses that demonstrated relatively poor infectivity when the intracellular domain of gp41 was derived from pNL4-3 did not show improved infectivity when the homologous intracellular domain of gp41 was used (e.g., the viruses with Env from patient 5 and the least infectious virus from patient 4). Indeed, the use of the homologous intracellular domain of gp41 led to a moderate loss in infectivity for the viruses from patient 4 and some viruses from patient 5, consistent with the possibility that incompatibilities existed in these cases between the gp41 sequences and the gag protein from pNL4-3.

### Infectivity of recombinant viruses carrying primary envelope sequences in MT4-R5 cells

**Figure 4:**
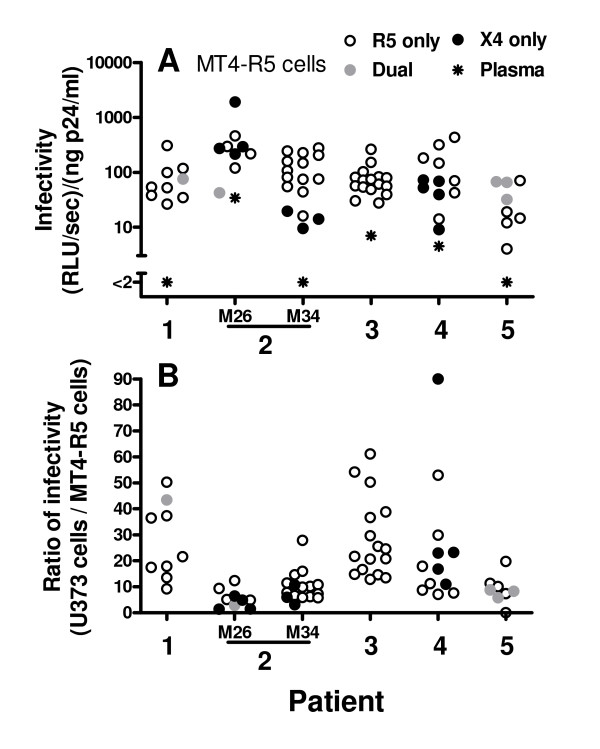
**Infectivity of recombinant viruses carrying primary Env sequences in MT4-R5 cells**. (A) Recombinant reporter viruses were generated as described in Fig. 1 legend, and the ability of these viruses to infect MT4-R5 cells was measured by evaluating luciferase expression in the target cells 44 hours after infection. For patient 2, clonal viruses were obtained from plasma samples obtained 8 months apart (26 and 34 months after initial diagnosis). The tropism of the recombinant viruses, as defined by their ability to infect U373-R5 and U373-X4 cells is shown: strict R5, open symbols; dual, solid gray symbols; strict X4, solid black symbols. Results for viruses expressing *env *sequences amplified by RT-PCR from patient plasma is also shown (stars). Each symbol is the mean of at least 3 independent experiments; the mean coefficient of variation for these results is 37% (range 1 – 78%). For each patient, significant differences were found comparing the viruses with the highest and lowest infectivity (p < 0.05 – 0.005 by t-test). (B) For each recombinant virus, the infectivity [(RLU/sec)/(ng p24/ml)] observed using U373 target cells and MT4-R5 target cells is expressed as a ratio.

For all 70 recombinant viruses, the amount of luciferase activity resulting from infection of U373 cells was greater than that seen after infection of MT4-R5 cells. For each patient, a significant correlation was observed between the infectivity of viruses with R5-exclusive tropism for U373-R5 and MT4-R5 target cells (p < 0.05 for all comparisons). As was observed when U373 cells were used as targets, viruses carrying different *env *sequences from the same patient showed considerable variability in infectivity when MT4-R5 cells were infected (Fig. 4A). The difference in infectivity between the most and least infectious R5 viruses from each of the 5 patients averaged 1.2 ± 0.31 log_10 _(range 0.8 – 1.5 log_10 _difference). As indicated above, the infectivity of viruses from patient 5 were strikingly lower than that of viruses from other patients when U373-R5 cells were used as targets. This difference was less striking when MT4-R5 cells were infected (compare Figs. 2A and 4A), although the infectivity of R5 viruses from patient 5 remained significantly lower than that of R5 viruses from patients 2 and 3 (p < 0.05 for both comparisons).

**Figure 5:**
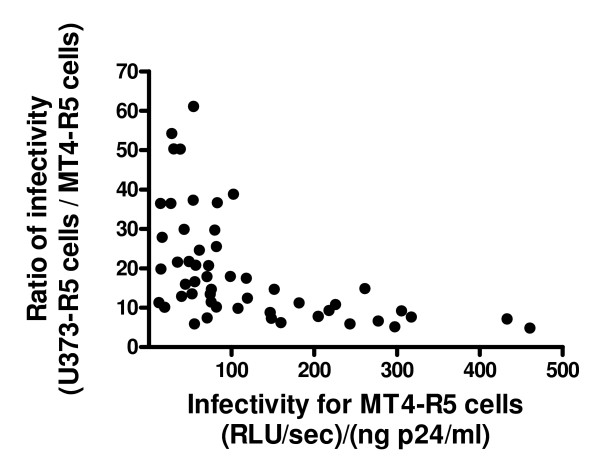
**Relationship between the infectivity of recombinant viruses bearing envelope proteins from plasma viruses for MT4-R5 cells and U373-R5 cells**. Recombinant reporter viruses were generated as described in Fig. 1 legend, and the ability of these viruses to infect MT4-R5 cells and U373-R5 cells was measured by evaluating luciferase expression in the target cells 44 hours after infection. For each of the 53 viruses with strict R5 tropism, the infectivity ratio (infectivity for U373-R5 cells/infectivity for MT4-R5 cells) is expressed as a function of the infectivity for MT4-R5 cells [(RLU/sec)/(ng p24/ml)]. A significant inverse correlation between these parameters was observed (Spearman r = -0.64, p < 0.0001).

**Figure 6:**
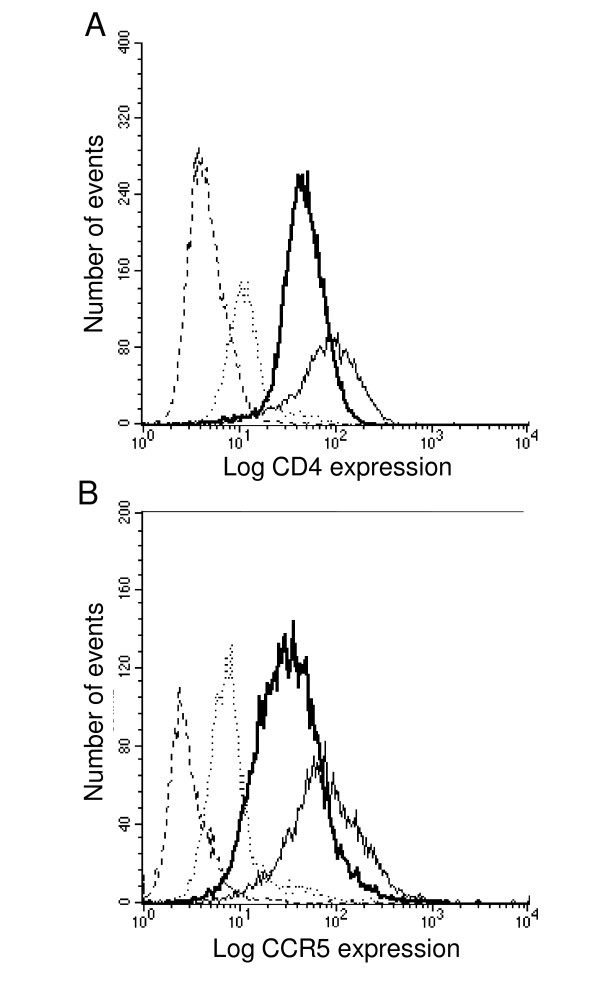
**Expression of CD4 and CCR5 on U373-R5 cells and MT4-R5 cells**. Cells were resuspended in PBS containing 20% human serum and incubated with directly-conjugated monoclonal antibodies or isotype-matched control antibodies conjugated with the same flurochrome. (A) CD4 expression on U373-R5 cells (thin solid line) and MT4-R5 cells (heavy solid line) detected using FITC-labelled mouse anti-human CD4 monoclonal antibody (clone RPA-T4). Binding of an isotype-matched control antibody conjugated with FITC is shown in the corresponding dashed lines. (B) CCR5 expression on U373-R5 cells (thin solid line) and MT4-R5 cells (heavy solid line) detected using phycoerythrin-conjugated mouse anti-human CCR5 monoclonal antibody (clone 2D7). Binding of an isotype-matched control antibody conjugated with phycoerythrin to these cells is shown in the corresponding dashed lines.

Interestingly, considerable variability was observed when luciferase activity obtained following infection of the two cell types was expressed as a ratio (Fig. 4B). This ratio did not correlate with the infectivity of the viruses for U373 cells (Spearman r = 0.10, p = 0.49), but only viruses with relatively low infectivity for MT4-R5 cells [i.e., <110 (RLU/sec)/(ng p24/ml)] had values for this ratio that were greater than 20 (Fig. 5). The level of expression of both CD4 and CCR5 was approximately two-fold higher on U373-R5 cells than on MT4-R5 cells (Fig. 6). Thus, a possible explanation for these observations is that the infectivity of viruses for which a high ratio was observed are particularly sensitive to the levels of expression of CD4 and/or co-receptor, although other differences between U373-R5 cells and MT4-R5 cells may also influence the ability of viruses carrying different envelopes to infect these cell types.

It is noteworthy that the proportion of viruses with a relatively high infectivity ratio was different in different patients. The infectivity ratios of clones from patients 1, 3 and 4 were significantly greater than that of patient 2 (p < 0.001, p < 0.001 and p < 0.05, respectively), and the infectivity ratio of clones from patient 3 was also significantly greater than that of patient 5 (p < 0.05).

### Infectivity of recombinant viruses carrying envelope sequences amplified from plasma by RT-PCR

In parallel with studies evaluating the infectivity of recombinant viruses carrying *env *sequences derived from clonal viruses, we evaluated the infectivity of recombinant viruses expressing *env *sequences amplified by RT-PCR from viral RNA extracted from the same plasma specimen from which the clonal viruses had been derived. The infectivity of viruses carrying plasma-derived *en*v sequences for U373-R5 cells, although detectable in all cases except patient 2, was generally low, and was less than that of the clonal viruses from the same patient, usually by a substantial margin (Fig. 2A). The infectivity of viruses expressing plasma-derived *env *sequences for U373-X4 cells was detectable in only two samples (Fig. 2B). In both of these cases (patient 2, M26 and patient 4), clonal viruses with X4 exclusive tropism had been identified. The failure to detect infectivity of viruses carrying plasma-derived *env *sequences in other samples from which clonal viruses with strict X4 or dual tropism were identified may reflect the somewhat lower infectivity of the viruses with X4 tropism (e.g., patient 2, M34 and patient 5) and/or a lower proportion of viruses with X4 tropism in the sample (e.g., patient 1). As was observed for viruses carrying *env *sequences derived from clonal viruses, the infectivity of viruses carrying plasma-derived *env *sequences for MT4-R5 cells was usually reduced compared to that observed for U373 cells (Fig. 4A). Indeed, for patients 1 and 5, low level infectivity was detected toward U373-R5 cells, but infectivity was below detection when MT4-R5 cells were targeted.

### Sensitivity of recombinant viruses carrying primary envelope sequences to entry inhibitors

**Figure 7:**
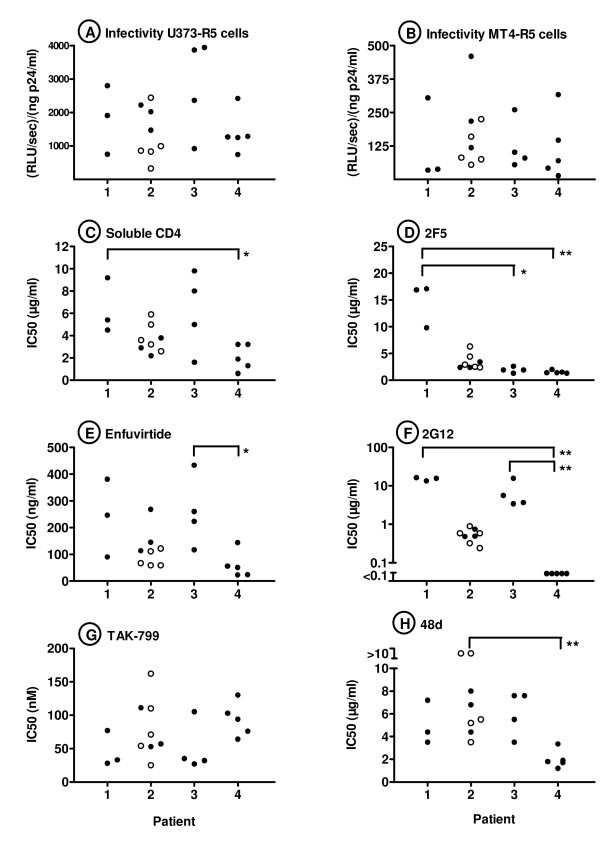
**Sensitivity of recombinant viruses carrying primary envelope sequences to entry inhibitors**. Clonal viruses with R5-tropism exhibiting a spectrum of infectivities towards U373-R5 cells (panel A) and/or MT4-R5 cells (panel B) were selected from among those obtained from patients 1–4. For each of these 20 viruses, the IC50s were determined on U373-R5 cells for soluble CD4 (C), enfuvirtide (E), TAK-799 (G), and neutralizing monoclonal antibodies 2F5 (D), 2G12 (F), and 48d (H). Each symbol represents the mean of three independent determinations. For all inhibitors except TAK-799, significant patient-specific differences in IC50 were observed using the Kruskal-Wallis test. Brackets indicate significant pairwise differences in post-test comparisons performed using Dunn's multiple comparison test (* p < 0.05; ** p < 0.01). For patient 2, clonal viruses were obtained from plasma samples obtained at both 26 months (solid symbols) and 34 months (open symbols) after initial diagnosis.

Because the infectivity of clonal viruses carrying different *env *sequences from a given patient varied over a wide range, these viruses were useful in exploring the possible relationship between infectivity and sensitivity to inhibition by entry inhibitors. To do so, clonal viruses with R5-tropism that exhibited a spectrum of infectivities towards U373-R5 cells and/or MT4-R5 cells were selected from 4 of the patients (Figs. 7A and 7B). For each of these 20 viruses, the IC50s were determined on U373-R5 target cells for two entry inhibitors (enfuvirtide and TAK-799), soluble CD4, and neutralizing monoclonal antibodies recognizing either gp120 (2G12, 48d) or gp41 (2F5). No significant correlations were observed between the IC50s of these six inhibitors and the infectivity of the clonal viruses for either U373-R5 cells or MT4-R5 cells (data not shown, p > 0.09 for all Spearman correlations).

Viruses from different patients did, however, demonstrate differential sensitivity to these entry inhibitors, independent of infectivity (Fig. 7). Thus, significant differences in the median sensitivity to entry inhibitors by clonal viruses from different patients was observed by ANOVA for all entry inhibitors except TAK-799 (p values for Kruskal-Wallis test: p < 0.001 – 0.05), and for each of these inhibitors, significant differences in were also identified in pair-wise comparisons of IC50s for viruses from different patients (Fig. 7).

### Genotype-phenotype correlations

Nucleotide sequences for *env *extending from the signal peptide to mid C4 region were available for all clones. The PSSM score developed by Jensen et al. [49] correctly distinguished all clones with R5-exclusive tropism and X4-exclusive tropism, even though viruses from two of the patients were non-B subtypes. Three of the 6 Env proteins with dual tropism, however, were predicted to exhibit R5-exclusive tropism (one clone each from patients 1, 2 and 5). The amino acid sequence of the V3 region of these dual-tropic clones from patients 1 and 2 differed at 6 and 1 positions, respectively, compared to that of the most similar R5-tropic Env identified in that patient, and these differences may explain the change in tropism. The V3 region of the misidentified dual-tropic envelope sequence from patient 5, however, was identical to that of other Env with R5-exclusive tropism, indicating that sequences outside V3 influenced the tropism of this Env.

**Figure 8:**
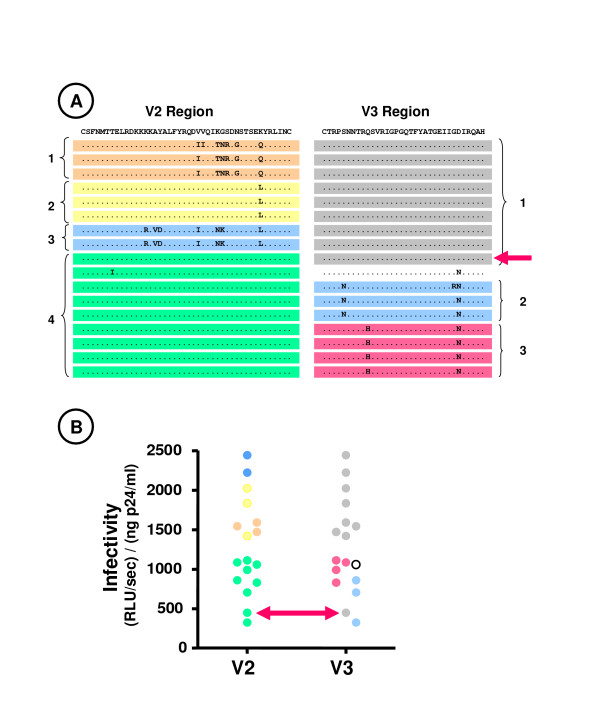
**Expression of V2 and V3 region haplotypes in Env proteins of clonal viruses from patient 2 and viral infectivity**. (A) The haplotypes in the V2 region (left) and V3 region (right) of the Env proteins expressed by the 17 clonal viruses from patient 2 with strict R5 tropism are shown. The consensus amino sequence is shown on the top line, and only amio acids differing from the consensus sequence are shown for each clone. For each variable region, sequences that are identical or that differ by a single amino acid substitution not identified in another sequence are highlighted by the same color, and these haplotypes are also identifed by numbers adjacent to the brackets. The red arrow indicates the envelope expressing the V2 region haplotype 4 associated with the V3 region haplotype 1 (see text). (B) The infectivity of recombinant viruses expressing these Env proteins using U373-R5 cells is shown. For each of the variable regions, the color of the symbols corresponds to that of the V region haplotype expressed by that virus. The red arrow indicates the infectivity of the clone expressing the V2 region haplotype 4 associated with the V3 region haplotype 1.

In general, viruses with strict R5-tropism from the same individual expressed a relatively small number of haplotypes within a given variable (V) region. For example, the number of distinct haplotypes identified for the V3 region ranged from 1 (patient 1) to 4 (patient 2). Significant differences in the infectivity of R5-tropic viruses as a function of haplotype were observed for the variable regions 2 and 3 (V2 and V3) of patient 2 (p < 0.02 and p < 0.03 respectively using the Kruskal-Wallis test). As shown in Fig. 8, the viruses from this patient expressing V3 region haplotypes 2 and 3 were significantly less infectious than those expressing haplotype 1 (p < 0.01 using the Mann-Whitney test), and viruses expressing the V2 region haplotype 4 were less infectious than those expressing the V2 region haplotypes 1 – 3 (p < 0.001). Most of the viruses expressing the V2 haplotype associated with low infectivity (haplotype 4) also expressed V3 haplotypes associated with low infectivity. However, one of the viruses expressed this V2 haplotype in association with the V3 haplotype 1, which was usually associated with good infectivity (red arrow in Fig. 8A). The infectivity of this virus was, nevertheless, low (red arrow in Fig. 8B), suggesting that expression of the V2 haplotype 4 was a major determinant for low infectivity in this patient. It is noteworthy that clones from patient 2 expressing the V2 haplotype 4 were obtained only from the plasma sample obtained at month 34, and 9/13 clones with R5-tropism isolated at this time point expressed this V2 haplotype.

No significant associations between infectivity and haplotype were observed for the variable regions expressed by the other patients, and no association between infectivity and the haplotypes in the constant regions were identified. One factor that could confound such analyses is the presence of deleterious mutations elsewhere in the envelope sequence. In this regard, we found that four of the 53 viruses with strict R5 tropism had V3 sequences containing a single amino acid polymorphism not seen in any other sequence from that patient. The recombinant viruses carrying 3 of these unique V3 polymorphisms had the lowest infectivity for U373-R5 cells of any virus evaluated from that sample.

## Discussion

In this study we have explored the functional properties of Env proteins from contemporaneous HIV-1 biological clones isolated from the plasma of chronically infected patients. Even when comparisons were restricted to viruses with similar tropism, these genetically diverse Env proteins were found to exhibit striking functional diversity, including, i) a wide range of infectivities for a given target cell, ii) differences in relative ability to infect different target cells, and iii) differences in sensitivity to certain entry inhibitors. In addition to the functional diversity observed among viruses from a single patient, significant inter-patient differences were also observed for some characteristics of Env proteins, including sensitivity to several different entry inhibitors and the relative ability of viruses to infect different target cells. Interestingly, no correlation was observed between viral infectivity and sensitivity to entry inhibitors, indicating that these properties are, to some extent, dissociable. These findings demonstrate the marked functional heterogeneity of HIV-1 Env proteins expressed by contemporaneous circulating viruses, and underscore the advantage of clonal analyses in characterizing such viral populations.

The observation that Env proteins expressed by contemporaneous viruses display a broad spectrum of infectivity towards a given target cell raises the question of the forces responsible for this functional diversity. One factor that can drive the development of diversity is the selection in a single patient of numerous viral subpopulations with distinct Env functional properties. Genetically distinct viral populations replicating in different tissues or cellular compartments have been identified in numerous studies [50-54]. Such populations can also result in functional diversity, as illustrated by the obvious example of the coexistence of viruses with different tropism. Indeed, we found that the coexistence of viruses with R5 and X4 tropism could make an important contribution to overall genetic diversity in patients from whom both populations were isolated.

The high mutation and recombination rates of HIV-1 are also likely to have an important impact on infectivity. Because of the numerous structural constraints in viral proteins, including Env, many mutations will prove to be deleterious for fitness. Similarly, recombination events that shuffle *env *segments can modify the function of Env proteins [55,56]. Indeed, genetic evidence supports the idea that purifying selection against deleterious mutations is occurring in the *env *region [57]. In this regard, we observed that 3 of 4 viruses in which V3 sequences contained a single amino acid polymorphism not seen in any other sequence from that patient had low infectivity. The association of such rare polymorphisms with poor infectivity is consistent with the possibility that the deleterious mutations contribute to the wide spectrum infectivity observed in these studies, although further studies are required to directly demonstrate the impact of such specific genetic differences on viral infectivity.

The extent that genetic differences deleterious for viral replication accumulate because they are associated with escape from immune responses is also an important issue. In this regard, we observed that a V2 haplotype associated with reduced viral infectivity emerged in patient 2 over an 8 month interval, and had become the dominant haplotype. This haplotype differed by a single amino acid change from another haplotype observed in this individual (Fig. 8). Such a modification would be typical of escape from neutralizing antibodies or cytotoxic T-cells targeting this epitope, although other explanations are also possible (e.g., adaptation of the Env proteins to improve the targeting of a specific cell type or fixation of a deleterious mutation through stochastic processes). Further studies comparing the spectrum of infectivity of Env proteins present in the genetically homogeneous viral populations of viruses present immediately before seroconversion with that observed following the development of the anti-viral immune response will help define the extent that selection of variants associated with immune escape affects Env function.

The possibility that viral evolution during in vitro culture contributed to diversity should also be considered. For example, greatest viral diversity was observed for patient 3, and clones from this patient emerged later than those from the other patients. However, control experiments (see Materials and Methods) showed no evidence for viral evolution during culture. In particular, the absence of polymorphic bases in the sequences of the viral clones indicates that variants were not emerging to the extent that they were detectable by bulk sequencing, and therefore were not sufficiently abundant to influence either viral diversity or the assessment of infectivity.

The spectrum of Env infectivity in vivo is almost certainly greater than observed in our study. The Env proteins studied by us were derived from viruses capable of productively infecting MT4-R5 cells. We must assume that the *env *sequences of these viruses were enriched for those with high infectivity in this cell system, and Env proteins carrying lethal mutations or mutations preventing efficient propagation in MT4-R5 cell cultures would not be sampled. We found that recombinant viruses produced using bulk *env *sequences amplified directly from plasma by RT-PCR had an infectivity that was lower than that of recombinant viruses carrying Env proteins from the clonal viruses derived from the same sample, as would be expected if a significant proportion of viruses present in plasma carry Env proteins that are noninfectious or whose infectivity is too low to permit emergence of clones during in vitro culture. The finding in previous studies that a substantial portion of viruses expressing *env *sequences amplified from plasma by RT-PCR show low or undetectable infectivity is also compatible with this interpretation [19-23,53,58]. It should be emphasized, however, that additional artifacts may also contribute to the low infectivity observed in our study for viruses carrying bulk *env *sequences amplified from plasma by RT-PCR, such that the infectivity of the recombinant viruses would not be reflective of viruses in plasma. First, recombinant viruses formed using mixtures of sequences amplified from plasma will express heterotrimeric Env proteins, including, for some samples, trimers containing sequences with both X4 and R5 tropism. In this regard, our preliminary results suggest that the infectivity of viruses generated using mixtures of clonal *env *sequences may be lower than the mean infectivity of viruses expressing each of these *env *sequences as homotrimers. In addition, during the amplification of *env *sequences from plasma, recombination between the heterogeneous target sequences can occur, and may form sequences in which incompatibilities between Env segments are deleterious to infectivity. In view of these uncertainties, further studies will be required to fully define the spectrum of infectivity of Env proteins expressed by viruses in plasma.

An interesting observation from our study was the absence of correlation between the infectivity of the recombinant viruses studied and their susceptibility to several different entry inhibitors or neutralizing antibodies. The affinity of Env-coreceptor interactions is one factor that can influence both Env fusion kinetics and the sensitivity of Env to the inhibition by enfuvirtide and TAK-779 [35,36,56,59], although mutations that modify sensitivity to co-receptor antagonists without modifying fusion kinetics have also been described [36,60]. The failure to find a correlation between Env infectivity and sensitivity to these inhibitors suggests that differences affecting membrane fusion that are independent of Env-coreceptor affinity (e.g., "fusogenicity") or differences affecting other Env properties (e.g., the expression or stability of Env trimers) make an important contribution to the wide spectrum of Env infectivities observed in this study.

We did observe, however, that sensitivity to inhibition by soluble CD4, enfuvirtide, and three different neutralizing antibodies were properties that were shared by viruses from a given patient, independent of their infectivity. Similarly, Ray et al. found differences in enfuvirtide sensitivity comparing Env clones isolated from different patients [58]. These findings suggest that genetic determinants important in defining the sensitivity to these entry inhibitors lie in regions that are not necessarily subject to extensive diversity. For example, determinants in the relatively well conserved membrane proximal ectodomain of gp41 appear to be important in determining sensitivity to both enfuvirtide and the monoclonal antibody 2F5 [61,62]. Determinants of sensitivity to inhibitors of coreceptor binding appear to be subject to greater intra-patient variability. No patient-specific differences in sensitivity to TAK-779 was observed in our study, and considerable variability in sensitivity of individual Env clones from a given patient has been reported for several other coreceptor antagonists [58]. Thus, the impact of viral diversity on sensitivity to entry inhibitors is likely to differ for inhibitors with different modes of action. Our findings suggest, however, that resistance to entry inhibitors is not likely to be a useful surrogate marker for viral infectivity.

## Conclusion

These studies highlight the difficulty in defining the replicative "fitness" of viral Env proteins from a given patient, because viruses can display a large spectrum of Env infectivities and this spectrum is different in different cell types. Similarly, our studies confirm that considerable variability can be encountered in the sensitivity of individual Env proteins to entry inhibitors, and that this parameter can vary independently of Env infectivity. Only through clonal analysis can the heterogeneity of these Env properties be fully appreciated. As discussed above, further characterization of the spectrum of Env infectivity at different stages of disease evolution should provide insights into factors that govern viral pathogenesis. Because these parameters may differ in different individuals, this information may prove to be of prognostic significance.

## Methods

### Patients

Clonal viral populations were obtained from five patients chronically infected with HIV-1. Clinical information of these patients is summarized in Table 1. All patients were evaluated at the Hôpital Bichat – Claude Bernard, and informed consent was obtained prior to participation in the study. Three of the patients were not receiving treatment with antiretroviral drugs at the time of initial evaluation (patients 1–3). Patient 1 had never been treated. Patient 2 had received intermittent treatment with several regimens containing nucleoside analog reverse transcriptase (RT) inhibitors and protease inhibitors over a two year period, but had discontinued therapy 2 months before evaluation. Patient 3 had been treated for 1 year with AZT+3TC+efavirenz, but treatment had been discontinued for 3 years prior to evaluation. Patients 4 and 5 had a ≥ 2 year history of treatment failure. Both had been treated initially with nucleoside analog RT inhibitors, and subsequently received regimens also including non-nucleoside RT inhibitors, protease inhibitors and/or enfuvirtide in various combinations, but plasma virus had never become undetectable. Neither patient had received antiviral agents that target viral entry for at least 1 year prior to evaluation. For patient 2, clonal viral populations were obtained from two different plasma samples obtained eight months apart.

**Table 1:** Clinical characteristics of patients at time of study and number of clonal viruses obtained

Patient	Age/Sex	Months since diagnosis	Treatment*	Viral Load (log_10_)	CD4 T-cells (cells/μl)	Number of clones	Time to emergence of clones (days)
1	35/M	17	none	5.52	276	9	18 – 26
2	38/M	26	none	6.67	7	9	11 – 20
		34	3TC ddI LPV ^#^	6.32	18	16	11 – 20
3	41/M	210	none	6.22	151	16	20 – 36
4	38/M	114	3TC SQV/RTV	5.43	11	12	14 – 27
5	40/M	139	3TC ddI TDF TPV/RTV APV	5.33	8	8	10 – 21

*Abbreviations for antiretroviral drugs are as follows: amprenavir (APV), didanosine (ddI), lamivudine (3TC), ritonavir (RTV), saquinavir (SQV), tenofovir (TDF), tipranavir (TPV). ^#^Poor compliance suspected.

### Clonal viral populations

Clonal viral populations were obtained as previously described [41,42]. Briefly, MT4-R5 cells, expressing CCR5 and CXCR4 receptors, were resuspended at 2 × 10^6 ^cells/ml in complete medium containing 1% (v/v) DMSO, and 0.25 ml aliquots were distributed in 24-well plates. An equal volume of plasma, diluted in complete medium containing (final concentration) 1% DMSO and 2 μg/ml DEAE-dextran, was added to each well. The plates were centrifuged (860 xg; 2 hours; 22°C), and cultured for 4 hours at 37°C to permit viral entry. Cells were recovered, washed once, and 200 μl aliquots containing 2 × 10^4 ^cells were distributed into 96-well plates. The cultures were maintained at 37°C in 5% CO_2_, and were passaged with a 1:10 dilution every 7 days. Cultures were inspected by light microscopy, and when patent cytopathic changes were observed, the culture supernatant and the cell pellet from infected wells were recovered separately and frozen at -80°C. If viral replication was observed in >20% of the wells, the experiment was repeated after further dilution of the plasma. The days in culture at which the clones from different patients emerged is shown in Table 1. Seventy-five percent of all clones had emerged by day 24.

Findings in this and our prior studies [41,42] supported the conclusion that viral evolution was not occurring during culture. In control experiments, pNL4-3-derived viruses containing protease mutations that substantially impaired viral fitness (replicative capacity 10–20% of wild-type virus) were used in our protocol to generate clonal viruses, and the protease region from 36 different clones was sequenced. Without exception, these sequences were identical to the original plasmid (14,256 bases) and no evidence of reversion of the deleterious mutations was observed, despite that reversion of any of the mutations would have substantially improved fitness. In addition, proviral DNA (including *env*) was amplified and sequenced for every clone used in our study, and without exception, no ambiguous bases were identified. The absence of detectable polymorphisms is consistent with the interpretation that variants were not emerging during culture to the extent that their presence was detectable by bulk sequencing (i.e., >10%).

### Vectors

We have previously described a pNL4-3-derived proviral vector in which the *env *sequence coding for most of gp120 and the ectodomain of gp41 (nucleotides 6480 to 8263) has been deleted and replaced by a linker sequence containing a unique MluI restriction site [34]. To create a similar vector expressing *Renilla *luciferase in place of Nef, the BamHI – NcoI fragment was removed, and the remaining fragment was ligated with the BamHI – KpnI fragment from pTN7-NL [63] and the KpnI – NcoI fragment from pNL4-3, creating (pNL4-3-ΔectoENV-lucR). An analogous proviral vector was also constructed in which all of the *env *sequence except for that coding the 13 N-terminal amino acids of gp120 and the 29 C-terminal amino acids of gp41 (nucleotides 6344–8691) has been deleted and replaced by a linker sequence containing a unique NheI restriction site (pNL4-3-ΔENV-lucR).

### Amplification of proviral DNA

DNA was extracted from the infected cell pellets using a QIAamp Viral DNA mini kit (Qiagen, Valencia, CA), resuspended in 100 μl of 10 mM Tris buffer containing 1 mM EDTA, and used as a template to amplify *env *fragments. To amplify the 2.2 kb fragment that spans the *env *region deleted from the pNL4-3-ΔectoENV-lucR vector and also containing 147 bp (N-terminal) and 258 bp (C-terminal) extensions to allow homologous recombination with this vector, reactions (50 μl) contained 2 μl DNA, 200 μM each dNTPs, 1.5 mM Mg^2+^, 0.5 μM each oligonucleotides E5 (5'-GTCTATTATGGGGTACCTGTGTGGA) and FuB (5'-GGTGGTAGCTGAAGAGGCACAGG), 1U Phusion hot start DNA polymerase (Finnzymes, Espoo, Finland), and 1X Phusion HF reaction buffer. Cycling conditions were as follows: 98°C for 30 sec, followed by 5 cycles at 98°C for 5 sec, 72°C for 5 sec and 72°C for 90 sec each; 5 cycles at 98°C for 5 sec, 70°C for 5 sec and 72°C for 90 sec each; 30 cycles at 98°C for 5 sec, 68°C for 20 sec and 72°C for 90 sec each; with a final step at 72°C for 10 min. PCR products were purified by QIAquick PCR Purification Kit (Qiagen). Aliquots of the purified DNA was electrophoresed into agarose gels and stained with ethidium bromide to verifiy that a single band of appropriate size had been amplified and to permit quantification of the product. The 2.6 kb fragment that spans the *env *region deleted from the pNL4-3-ΔENV-lucR vector and also comprises 143 bp (N-terminal) and 104 bp (C-terminal) extensions to allow homologous recombination with this vector was amplified using similar conditions, except that oligonucleotides EB1 (5'GAAAGAGCAGAAGACAGTGGCAATGA) and EB2 (5'-ACTTGCCACCCATCTTATAGCAAA) were used, and cycling conditions were: 98°C for 30 sec, followed by 40 cycles at 98°C for 5 sec, 70°C for 20 sec and 72°C for 90 sec each; and a final step at 72°C for 10 min.

To amplify *env *sequences present in plasma, RNA was isolated from plasma using a QIAamp RNA Blood Mini Kit (Qiagen), and cDNA was synthesized using SuperScript III reverse transcriptase (Invitrogen, Carlsbad, CA) and random hexamer primers. *Env *sequences were amplified in a single reaction using the protocol described above.

### Cell culture

293T cells and U373-CD4 cells were cultured in Dulbecco's modified Eagle medium supplemented with 10% fetal calf serum, 100 U/ml penicillin G and 100 μg/ml streptomycin (complete medium). MT4-R5 cells were cultured in similarly supplemented RPMI-1640 medium. For U373-CD4 cells stably expressing CCR5 or CXCR4 [64], medium also contained 10 μg/ml puromycin and 100 μg/ml hygromycin B.

### Production of recombinant viruses

The techniques used to produce recombinant viruses have previously been described [34,65,66]. Briefly, 293T cells that had been grown to 80% confluency in T25 flasks were co-transfected with 8 μg of MluI-linearized pNL4-3-ΔectoENV-lucR vector (or NheI-linearized pNL4-3-ΔENV-lucR vector) and 1 μg of the corresponding PCR product using the calcium phosphate precipitation method, and cultured in 3 ml of complete medium. After 18 h, cells were washed with phosphate-buffered saline (PBS) and culture medium was replaced. Forty-four h after transfection culture medium was recovered and centrifuged (800 xg; 10 min), and p24 antigen present in the supernatant was measured using an enzyme-linked immunosorbent assay (Innotest HIV antigen mAB, Innogenetics, Gent, Belgium).

### Evaluation of viral infectivity

The infectivity of recombinant viruses for different target cell types was determined by measuring luciferase activity. To do so, target cells were plated in black-wall, clear bottom 96-well plates (Greiner, Courtaboeuf, France). U373 cells were plated at 2 × 10^3 ^cells/well 48 h prior to infection; MT4-R5 cells were plated at 5 × 10^4 ^cells/well on the day of infection. Medium was removed and replaced with 60 μl of complete medium containing serial two-fold dilutions of freshly harvested supernatants from transfected cells containing 0.069 – 50 ng p24/ml. Forty-four hours after infection, 20 μl of 4X luciferase lysis buffer (Renilla luciferase assay system, Promega, Charbonnières, France) was added to each well, and plates were maintained at room temperature for 30 min. Wells were sequentially injected with 100 μl of luciferase substrate (Promega), and 3 seconds later, light emission (relative light units, RLU) was measured over a two sec interval using a Microlumat LB96P luminometer (Berthold, Oak Ridge, TN). Each sample was evaluated in triplicate. RLU were plotted as a function of amount of p24 used to infect the cells, and infectivity was defined as the slope [(RLU/sec)/(ng p24/ml)] as determined by linear regression; in this analysis, each replicate RLU value was treated as an individual point.

In preliminary experiments, we compared the infectivity of viruses obtained after transfection of 293T cells with proviral plasmids, and viruses created by recombination between *env *sequences amplified from the same plasmid and the pNL4-3-derived ΔEnv vector according to our protocol. The infectivity of the viruses produced through recombination was 89 ± 37% (mean ± SD; n = 5) of that observed for viruses obtained directly by transfection (p > 0.2 by paired t-test).

For each viral Env protein studied, infectivity was determined for each cell type in at least three independent experiments. Infection was considered undetectable if the RLU observed for the highest concentration of p24 evaluated was less than 500 RLU/sec (i.e., infectivity <2 (RLU/sec)/(ng p24/ml). The mean coefficient of variation for clonal Env proteins with detectable infectivity was as follows: U373-R5 cells (n = 58), 42%; U373-X4 cells (n = 17), 50%; MT4-R5 cells (n = 70), 37%.

### Inhibition of infectivity by entry inhibitors

To measure the susceptibility of recombinant viruses to inhibition by enfuvirtide and TAK-799, U373-R5 cells were plated at a density of 6 × 10^3 ^cells/well as described above, and infected with the same dose of recombinant virus in the absence or the presence of increasing concentrations of enfuvirtide (1.6 to 5,000 ng/ml; T20, American Peptide Company, Sunnyvale, CA) or TAK-799 (0.8 to 2500 nM; NIH AIDS Research and Reference Reagent Program). Forty four hours after infection, luciferase activity was measured as described above. For each virus, an amount of p24 was used that gave approximately 5 × 10^4 ^RLU/second for cells infected in the absence of inhibitor. Each experimental condition was evaluated in triplicate. To determine the concentration of inhibitor required for reduce viral infectivity by 50% (IC50), data was fitted to a sigmoid dose-response curve with variable slope.

The susceptibility of recombinant viruses to inhibition by soluble CD4 (R&D Systems, Minneapolis, MN) and monoclonal antibodies 48d (human anti-gp120, NIH AIDS Research and Reference Reagent Program), 2G12 (human anti-gp120, Polymun Scientific, Vienna, Austria) and 2F5 (human anti-gp41, Polymun Scientific) was measured by similar techniques, except that recombinant viruses were preincubated for 1 h at 37°C with serial three-fold dilutions of soluble CD4 or monoclonal antibodies, before using 60 μl of the mixture to infect target cells. For each viral Env, infectivity was determined for each inhibitor cell type in at least two, and usually three independent experiments. The mean coefficient of variation for the six entry inhibitors ranged from 25% (enfuvirtide) to 64% (soluble CD4).

### Cytofluorometry

U373 cells were detached by incubation in PBS containing 0.8% EDTA. Cells were resuspended in PBS containing 20% human serum (1 × 10^6 ^cells; 100 μl) and 10 μl of one of the following monoclonal antibodies: phycoerythrin-conjugated mouse anti-human CCR5 (clone 2D7), phycoerythrin-conjugated mouse anti-human CXCR4 (clone 12G5), FITC-conjugated mouse anti-human CD4 (clone RPA-T4), or similarly conjugated isotype-matched control antibodies (BD Biosciences, San Jose, CA). Following a 1 h incubation at 4°C, cells were washed once and analyzed using a FACSCalibur flow cytometer (BD Biosciences). Analysis was restricted to viable cells, identified on the basis of forward and 90° scatter.

### Data analysis

Results are expressed as mean ± SD unless otherwise indicated. Comparisons between groups were performed using the Kruskal-Wallis test. Post test comparisons, performed only if p < 0.05, were made using Dunn's multiple comparison test. Correlations were evaluated using the Spearman test. Nucleotide diversity was determined using the method of Tajima and Nei [67].

## Competing interests

The author(s) declare that they have no competing interests.

## Authors' contributions

TN, FB performed the experiments and contributed to the analysis of data and writing the manuscript. BL, FM designed and constructed the vectors used and participated in design of the studies. CC contributed to obtaining the viral clones and the genetic studies. FC, AJH participated in the design of the study, the analysis of data and wrote the manuscript. All authors read and approved the final manuscript.
